# The effectiveness of an exergame intervention for college students with subthreshold depression: protocol for a mixed methods study

**DOI:** 10.3389/fpubh.2024.1390806

**Published:** 2024-09-02

**Authors:** Kexin Huang, Min Li, Simiao You, Yongliang Jiao, Rendong He, Bingyue Han, Yuhang Pu, Yong Jia, Li Chen

**Affiliations:** ^1^School of Nursing, Jilin University, Changchun, China; ^2^Invasive Technology Nursing Platform, The First Hospital of Jilin University, Changchun, China; ^3^School of Sport Health and Technology, Jilin Sport University, Changchun, China; ^4^Department of Psychiatry, University of Cambridge, Cambridge, United Kingdom

**Keywords:** subthreshold depression, college students, exergame, protocol, mixed methods intervention trial

## Abstract

**Background:**

Subthreshold depression (StD) is a condition that significantly influences the mental health and quality of life of college students and increases the risk of developing major depressive disorder (MDD). Exercise therapy has been found to be effective, but may not be enjoyable for everyone. exergames, as a form of exercise therapy, address the limitations of traditional exercise by incorporating gaming elements to make physical activity more entertaining and interactive. Currently, the Nintendo Switch is one of the most widely used exergame devices.

**Aims:**

To explore the effectiveness of a Nintendo Switch-based exergame intervention on college students with StD compared to a control group, and to analyze their perceptions of the program.

**Methods:**

This study will employ an explanatory sequential design, starting with a quantitative evaluation using a randomized controlled trial (RCT), followed by a supplementary qualitative study. College students identified as having StD will be randomly allocated in a 1:1 ratio into the exergame intervention group (EIG) or the control group (CG). College students in the EIG will participate in a Nintendo Switch-based exergame program for 8 weeks, with 2–3 sessions per week, lasting 50–60 min each. Participant outcomes in both conditions will be assessed at pre-intervention (T_0_, week 0), post-intervention (T_1_, week 8), 1 month after the intervention (T_2_, week 12), and 2 months after the intervention (T_3_, week 16), and a generalized linear mixed model will be used for analysis. In the qualitative part of this study, interviews will be conducted with college students with StD from the EIG at T_1_ to explore their experiences of receiving the intervention, and content analysis will be applied to the data collected.

**Discussion:**

Nintendo provides a user-friendly platform for college students with StD to engage in electronic gaming. Limited research has explored the mental health outcomes of interventions using this type of technology in young people with StD. If the exergame program proves to be effective, it could offer a convenient and feasible intervention for further enhancing the psychological well-being of college students.

**Clinical trial registration:**

This study was registered in the Chinese Clinical Trial Registry (number: ChiCTR2300068970) on 2nd March 2023.

## 1 Introduction

Subthreshold depression (StD), considered to be the precursor stage of major depressive disorder (MDD) (1), is defined as the simultaneous presence of 2 to 4 depressive symptoms for at least 2 weeks causing significant functional impairment in daily activities or social relationships, without meeting the full diagnostic criteria for a depressive disorder (2). The prevalence of StD ranges from 1.50% (3) to 41.27% (4) in adults. In college students, a prevalence of 40.8% has been reported (5), significantly exceeding the one for the general population (6). In this population, StD has been associated with different factors including interpersonal difficulties, and academic and employment pressures (6). StD can significantly impact the mental health and social functioning of the affected individuals (7), and has been linked with an increased risk of suicide (8).

Previous studies have reported an association between StD and adverse clinical outcomes including MDD, neurotic symptoms, and geriatric patients. Lyness et al. (9) conducted a cohort study including 622 elderly patients, and found that individuals with StD had a 5.5-fold increased risk of developing major depression 1 year after baseline evaluation, compared with non-depressed patients. An et al. (10) found that individuals with StD scored significantly higher in obsessive-compulsive disorder and anxiety. Goldney et al. (11) found that StD can lead to sexual dysfunction and suicide, resulting in a decline in the quality of physical, psychological and social relationships (12). In 2022, the Ministry of Education of the People’s Republic of China further emphasized the need to strengthen students’ mental health education, and to improve identification and prevention (13). Therefore, identifying effective interventions for StD to prevent its development into MDD has become a major public health priority.

In many countries, the management and intervention approaches for StD typically involve a comprehensive, multidisciplinary, and multifaceted treatment approach, encompassing both pharmacological interventions (PIs) and non-pharmacological interventions (NPIs). Currently, pharmacological interventions specifically tailored for StD are scarce. Although certain studies have demonstrated that antidepressants can mitigate depressive symptoms in adults with StD (14), there is not enough evidence to recommend the widespread use of antidepressants for the treatment of StD (15). In addition, a recent study has shown a significant effect of NPIs in preventing the onset of MDD and improving depressive symptoms (7). Exercise therapy, recommended in the UK’s National Institute for Health and Care Excellence (NICE) guidelines (16, 17), is characterized by lower costs, fewer side effects and rapid effectiveness compared to other treatments, and has been shown to be effective in reducing depressive symptoms (18). However, some studies suggest that traditional exercise can be considered monotonous and insufficiently stimulating, often lacking variety, reducing individuals’ willingness to participate and adhere in the long-term (19).

Exergames, a type of exercise therapy, overcome the limitations of traditional exercise therapy by incorporating gaming elements. By providing more stimulating and personalized exercise options, exergames enrich the forms and contents of exercise with fun, interaction, and pleasure, enhancing individuals’ motivation to participate in physical activity and improving the effectiveness of exercise in promoting health (20). Nintendo is one of the most popular tools in neural rehabilitation, favored for its unique interactivity and somatosensory controllers, and has been found to be safe (21, 22), useful (22–24), and feasible (22), as well as enjoyable and motivating (21, 22, 24). Studies have shown that Nintendo-based exergames can significantly improve depressive symptoms in older adults (25, 26), but there is little evidence in college students, who represent a vulnerable population. Although a study developed in China reported that exergames (27) could improve the depression in college students, the exergames were limited to a simple combination of sports and games.

Meanwhile, an in-depth understanding of the experiences of individuals with StD participating in exergames will help improving and promoting these intervention programs, as well as encouraging their use and acceptability. Wingham et al. conducted a qualitative study exploring the acceptability of using the Nintendo Wii Sports games in stroke survivors and showed the Wii was acceptable to these patients in home-based rehabilitation (28). Glännfjord et al. examined perceptions of using Wii Sports Bowling by older adults, who described it as enjoyable and a social activity (29). Overall, research exploring the experiences of using exergames in college students with StD is insufficient.

The main aim of this study is to explore the therapeutic effects and the experiences of using exergames in college students with StD. An explanatory sequential design will be used, starting with a randomized controlled trial (RCT) investigating the impact of a Nintendo Switch-based exergame intervention on mental health outcomes (depression, anxiety, sleep quality) in a group of college students with StD. For this, a personalized exergame program based on the characteristics, needs and preferences of college students with StD will be considered to tailor the intervention to their unique needs and stimulate their engagement. Subsequently, qualitative surveys will be conducted to gain an in-depth understanding of college students’ subjective experiences of participating in this exergame intervention, in particular the benefits arising from the intervention as well as the influencing factors for participation, which will help understanding the mechanisms behind the therapeutic effects. This information will provide a basis for optimizing, refining, and promoting exercise game intervention programs.

We hypothesize that participants in the exergame intervention group (EIG) will experience an improvement of depressive and anxiety symptoms, and sleep quality, compared to those in the control group (CG). Furthermore, we anticipate that these improvements will be maintained at 2-months follow-up.

## 2 Methods

### 2.1 Study design

**Figure 1:**
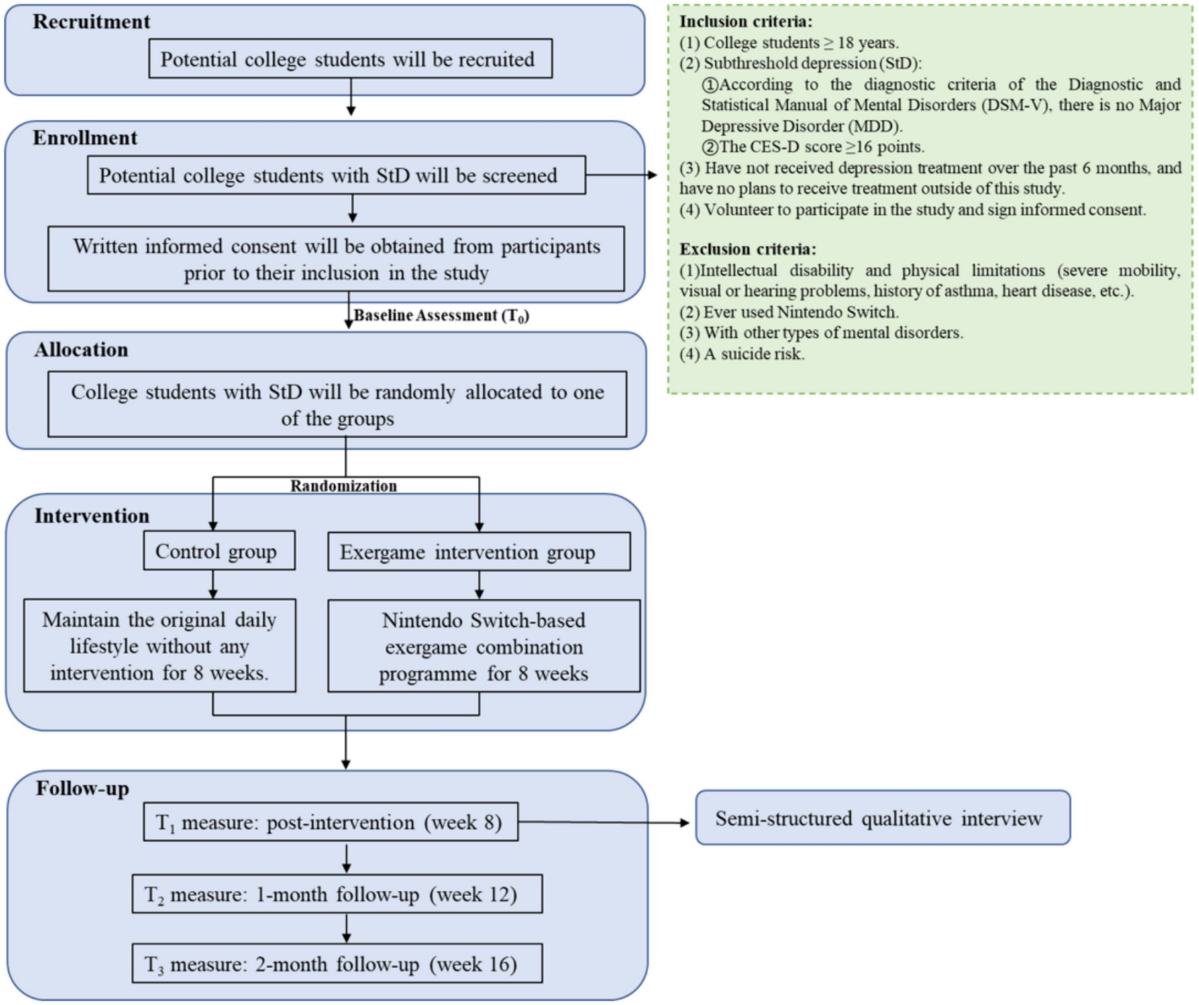
Study design flowchart.

This mixed methods study will comprise an assessor-blinded, parallel-group, randomized controlled trial and a qualitative study. Such a design is supported by the Medical Research Council’s guidance for evaluating complex interventions (30). The study will employ an explanatory sequential design, starting with a quantitative outcome-based evaluation exploring treatment effects, and followed by a qualitative study to gain insights into participants’ perceptions of the treatment. The qualitative study will allow understanding the real experiences of students with StD participating in the intervention and the mechanisms behind the treatment (31). The study design is illustrated in Figure 1.

### 2.2 Sample size and power calculations

For the quantitative part of this study, an *a priori* power analysis was conducted using G*power 3.1.9.7. A recent meta-analysis found a significant effect of exergame-based exercise training on depressive symptoms, with a moderate effect size of 0.69 (19). Assuming an attrition rate of 10%, a sample size of 76 participants (38 participants per arm) will be required to provide an 80% power to detect an effect size of at least 0.69 at a 5% significance level in a 2-arm trial.

For the qualitative component of this study, purposive sampling will be used to select college students with StD who completed the 8-week exergame intervention (excluding dropouts). The number of interviews will be determined by data “saturation” (32).

### 2.3 Eligibility and recruitment

Participants will be recruited from colleges in Changchun, in the Jilin Province in China. Students will be recruited through live lecture, poster promotion and official account by researchers and staff. Eligible subjects will be screened through a combination of face-to-face surveys and a QR code through the We-Chat Star questionnaire. Inclusion criteria for participants will be: (1) college students aged 18 years or older; (2) individuals meeting the diagnostic criteria for StD (they will not meet the diagnostic criteria for MDD according to the Diagnostic and Statistical Manual of Mental Disorders (DSM-V) (33) and their Center for Epidemiologic Studies Depression Scale (CES-D) score will be 16 or higher); (3) individuals who have not received depression treatment in the past 6 months and have no plans to receive treatment outside of this study; and (4) individuals who have volunteered and agreed to participate in the study and signed informed consent. Participants will be excluded if: (1) they present an intellectual disability and/or physical limitations (such as severe mobility, visual or hearing impairments, history of asthma, heart disease, etc.); (2) they have prior experience using the Nintendo Switch (to ensure consistency or comparability in gaming experience among the participants); (3) they present other types of mental disorders (to ensure research focus, accuracy, safety, and ethical compliance); and (4) they present a suicide risk (to ensure safety and ethical compliance of participants).

### 2.4 Randomization, allocation and blinding

Participants will be randomly allocated to the intervention or control groups in a 1:1 ratio using computer-based permuted block randomization. The randomization sequence will be generated by an independent research coordinator, and the details of group allocation will be concealed on cards placed inside sequentially numbered, sealed opaque envelopes. Outcome evaluators, data analysts and study coordinators will be kept blinded.

### 2.5 Interventions

#### 2.5.1 Pre-experiment

Before the pre-experiment, participants will be involved in two exergame practice sessions, lasting 60 min each. The pre-experiment will be conducted using a 2-week exergame program (test version; Supplementary Table S1), including 2–3 sessions per week (34), lasting 50–60 min each. During the first week of the exergame program, participants’ heart rates will be monitored using smart sports bracelets, and their maximum heart rate (HRmax) will be estimated using the formula HRmax = 207–0.7 × age to inform subsequent exercise intensity. The Physical Activity Rating Scale-3 (PARS-3) will be used to understand participants’ athletic abilities and preferences for the type of exercise. Additionally, the Favorite Exergame Questionnaire (FEQ) developed by our team and based on the “Fitness Ring Adventure” game, will be used to assess participants’ levels of interest, satisfaction, and enjoyment for the exergames (see Supplementary Table S2). Following this, a Nintendo Switch-based exergame combination program (preliminary version) will be set up, including “Warm-up,” “Exergame” and “Cool-down” phases. The program will be designed based on the information gathered and the exergame menu (see Supplementary Table S3).

The second week will be devoted to establishing the game plan (preliminary version), with the same intervention duration and frequency as in the first week. The intervention program will be adjusted according to the participants’ feedback and the recommendations of psychiatric nursing experts and sports training experts. For example, the content of the exercise game should progress from easy to difficult, be adjusted according to the participant’s interest, satisfaction, and enjoyment, and be diversified to include more game content, to increase interest and motivation for participation. The above information will result in a final Nintendo Switch-based exergame combination program (see Table 1 for an example of Week 1 and Supplementary Table S4 for the remaining 7 weeks).

**Table 1:** The Nintendo Switch-based exergame combination program.

Intervention time		Intervention content		Duration
Week 1	Warm-up		Dynamic Stretch Arm stretch (raise both arms and push forward) Abdominal stretch (swing arms, raise both arms and lean sideways) Leg stretch (step, deep squat) Yoga stretch (twisting seated pose)	5.5 min
	Exergame	Match1^a^	Adventure mode	30–40 min
		Match2^b^	Chest muscle training, Whack-a-mole Parachuting Squat jumps Icino land	10 min
	Cool-down		Stretching exercises^c^	4.5 min

^a^After each workout, keep a record, and finally accumulate and record the total score. ^b^Choose 1–2 challenges provided for completion. ^c^Stretching exercises focus on relaxing the abdominal, lumbar, and leg areas. mins, minutes.

#### 2.5.2 Formal intervention

##### 2.5.2.1 Intervention group

Before the intervention, two practice sessions will be conducted, lasting 60 min each. In the first session, a trained researcher will explain and demonstrate how to operate the equipment and will guide the participants through the exercises to familiarize them with the operation process and methods. In the second session, two researchers will assess participants’ proficiency in using the system and provide individualized guidance to ensure that all participants fully understand the system’s operation process and methods. In addition, at the end of the session, participants will receive a video recorded by the researcher demonstrating the operation procedures and methods, allowing those who may need additional time to familiarize themselves with the system.

The intervention group will be involved in the study for a total of 16 weeks, consisting of an 8-week intervention period and an 8-week follow-up period. The EIG will take part in the Nintendo Switch-based exergame combination program in an appointed psychological laboratory. Researchers will conduct the one-on-one intervention for 8 weeks, with 2–3 sessions per week, lasting 50–60 min each. Each session will include three phases: “Warm-up,” “Exergame” and “Cool-down” (see Supplementary Table S5, Figure S1 for details). To ensure game compliance and increase participation, the content of the program design will be different every week. The content of the “Cool-down” phase will be adjusted every 2 weeks, and the content of the exergame “Match 2” will be adjusted every week, and its difficulty and intensity will be increased gradually. Every Sunday, the week’s “Match 1” and “Match 2” results will be announced, and the following week’s game content will be released.

During the intervention, the researchers will keep an eye on the participants’ state to ensure the daily safety of the students.

##### 2.5.2.2 Control group

Participants in the control group will maintain their original lifestyle and will not receive any other intervention.

### 2.6 Outcome measures

#### 2.6.1 Quantitative outcome measures

Students both in the intervention and the control groups will be assessed on their levels of depressive and anxiety symptoms, sleep quality, participation adherence, and quality of life at T0, T1, T2 and T3. See Table 2 and Supplementary Figure S2 for a detailed description of data collection.

**Table 2:** Overview of data collection.

Measures	T0	T1	T2	T3
Demographics	△			
CES-D	△	△	△	△
PHQ-9	△	△	△	△
GAD-7	△	△	△	△
PSQI	△	△	△	△
SF-12	△	△	△	△

T_0_, pre-intervention (week 0); T_1_, post-intervention (week 8); T_2_, 1-month follow-up (week 12); T_3_, 2-month follow-up (week 16); CES-D, Center for Epidemiologic Studies Depression Scale; PHQ-9, Patient Health Questionnaire-9; GAD-7, Generalized Anxiety Disorder-7; PSQI, Pittsburgh Sleep Quality Index; SF-12, 12-Item Short Form Health Survey.

##### 2.6.1.1 The center for epidemiologic studies depression scale

Depressive symptoms will be measured using the Center for Epidemiologic Studies Depression Scale (CES-D), developed by Radloff et al. and revised by Zhang et al. (35, 36). It consists of 20 items using a 4-point Likert scale, ranging from “rarely or none of the time (less than 1 day)” to “most or all of the time (3–4 days).” The total score ranges between 0 and 60, with higher scores indicating greater levels of depression. A Cronbach’s α coefficient of 0.90 has been reported (35).

##### 2.6.1.2 The patient health questionnaire-9

The PHQ-9 (37) contains nine items, including loss of pleasure, low mood, sleep difficulties, lack of energy, eating disorders, low self-esteem, concentration difficulties, slow movement, and self-harm/suicidal ideas. These items use a 4-point Likert scale, ranging from “not at all” to “nearly every day.” The total score ranges from 0 to 7, with a higher score indicating a higher severity. This scale had a satisfactory Cronbach’s α coefficient among students (38, 39) and the internal consistency of the scale was also good.

##### 2.6.1.3 The generalized anxiety disorder scale

Anxiety symptom severity will be measured using the GAD-7, which consists of 7 items using a 4-point Likert scale, ranging from “Not at all” to “Nearly every day.” The total score ranges from 0 to 21, and scores of 5, 10, and 15 represent cut-points for mild, moderate, and severe anxiety, respectively. The scale has a Cronbach’s alpha coefficient of 0.92 and a retest reliability coefficient of 0.83 (40).

##### 2.6.1.4 The Pittsburgh sleep quality index

Sleep quality will be measured using the PSQI (41), which consists of seven subcategories: subjective sleep quality, use of sleeping medication, sleep latency, sleep disturbances, sleep duration, daytime dysfunction, and habitual sleep efficiency. The questionnaire consists of open-ended questions and 4-point Likert items, ranging from “Not during the past month” (0) to “Three or more times a week” (3) or from “Very good” (0) to “Very bad” (3), with higher scores indicating worse sleep quality. Lu et al. (42) reported a test–retest reliability of 0.994, a split-half reliability coefficient of 0.824, and an overall Cronbach’s alpha coefficient of 0.845 for the PSQI. In addition, the structural validity of the PSQI was 0.76, and the categorical validity was 0.81, indicating good reliability and validity (42).

##### 2.6.1.5 The 12-item short form health survey

The SF-12 (43) consists of 12 items measuring 8 health domains. It can be divided into a Physical Component Summary (PCS) and a Mental Component Summary (MCS). PCS includes Physical Functioning (PF), Role Physical (RP), Bodily Pain (BP), and General Health (GH) items. MCS includes Vitality (VT), Social Functioning (SF), Role Emotional (RE), and Mental Health (MH) items. The total score ranges from 0 to 100, with higher scores indicating better physical and mental health functioning. A score of 50 or higher is considered within the normal range.

##### 2.6.1.6 Adherence

The intervention was deemed successful if participants achieved at least 80% of the total target practice time (24). Based on an intervention design of 2–3 sessions per week for a period of 8 weeks, with each session lasting 50–60 min, the target practice time per week ranges from 100 (50 min × 2 times) to 180 (60 min × 3 times) minutes, the average target practice time per week is 140 min ((100 min + 180 min)/2), and the total target practice time is 1,120 min (140 min/week × 8 weeks). 80% of the total target practice time corresponds to 896 min (1,120 min × 80%). During the 8-week intervention, if the actual practice time of each participant reaches or exceeds 896 min, the completion standard is met.

#### 2.6.2 Qualitative data collection

The college students with StD who have completed the 8-week Nintendo Switch-based exergame intervention will be invited to participate in one-on-one, face-to-face semi-structured interviews to explore their experiences of taking part in the intervention. Table 3 shows the details of the interview outline.

**Table 3:** Semi-structured interview outline.

Number	Questions
1	What was your initial reason (or motivation) for participating in the exergame intervention? Please describe your reasons in order of importance.
2	What are your feelings after attending the exergame intervention?
3	What challenges have you encountered when participating in the exergame intervention? What was the biggest challenge? How was it resolved? Please provide examples and details.
4	Would you like to participate in other activities similar to the exergame intervention in the future? Would you recommend the exergame intervention to other people? Please state specific reasons.
5	What traditional exercise activities have you participated in before? In what ways do you think this combination of exercise and play could benefit you more than traditional exercise methods?
6	What kind of support and assistance in the exergame intervention would you like to receive in the future? Please be specific about what you think needs to be improved and refined.
7	Is there anything else you would like to add to today’s conversation?

### 2.7 Quality control

Our team is an experienced group of researchers, consisting of two senior experts in the fields of mental health and nursing and three graduate students specializing in nursing. If there are complex clinical issues, the researchers in our team and professional experts in the field of clinical psychiatry will work together to find solutions.

The researchers will provide uniform and standardized training to all the personnel responsible for implementing the intervention. This training will be conducted in a group and the use of the scales will be demonstrated and assessed to ensure that the implementers are familiar with both the content and use of the scales. In addition, testing and collection of questionnaire responses will be conducted on the spot to ensure the validity and recall of the questionnaire.

### 2.8 Statistical analysis

For quantitative analysis, Epidata 3.1 (Epidata Association) will be used for double data entry and SPSS 26.0 (International Business Machines Corporation, IBM) will be used for data analysis and processing. Descriptive statistics will be used to describe participants’ characteristics and mental health outcomes. For continuous variables, the Shapiro–Wilk test (S-W test) will be used to assess normality. Depending on the results of the S-W test, the mean and standard deviation or the median and the interquartile range will be used for descriptive statistics. For categorical variables, frequencies and percentages will be reported. Independent samples t-tests, Mann–Whitney U tests and χ^2^ tests will be used to compare outcome results and baseline data. Paired sample t-tests or non-parametric rank sum tests will be used to compare the outcomes within the group. To analyze between-group differences, a two-independent sample t-test or a Mann–Whitney U rank sum test will be used. The changing trend of the outcome indicators of the study subjects will be measured between T_0_, T_1_, T_2_, and T_3_; the inter-group factor will be the subgroup (EIG and CG), the intra-group factor will be the measurement time point (T_0_, T_1_, T_2_, and T_3_), and the interaction will be the group × time point. A generalized linear mixed model will be used for analysis. The significance level of all the above statistical tests will be set at *p* ≤ 0.05, indicating a significant statistical difference.

Regarding qualitative data analysis, one researcher will transcribe the interview audio recordings within 24 h after the interview and enter the transcriptions into Nvivo 12 (QSR International Pty Ltd), and another researcher will checked these transcriptions. Nvivo 12 will be used for coding, and data will be analyzed and collected in parallel. Qualitative content analysis will be applied to the data collected (44, 45). This method is an objective, systematic and quantitative research method applied to text content, which is based on the generation of explicit and descriptive content categories and the generation implicit and explanatory content themes. First, researchers will read the transcripts multiple times to get a sense of the data as a whole. Secondly, a series of open codes will be identified, and similar and related codes will be classified into subcategories. These subcategories will be then abstracted into generic categories, and finally, general categories will be summarized into main categories. Two researchers will analyze the data at the same time and compare their coding frameworks. If there is a disagreement, a third researcher will be consulted until an agreement is reached to improve the quality of the analysis.

### 2.9 Ethical approval and trial registration

The research protocol followed the SPIRIT (Standard Protocol Items: Recommendations for Interventional Trials) guidelines (46), has been reviewed and approved by the clinical research ethics committee of the School of Nursing of Jilin University (number: 2022091401), and has been registered in the Chinese Clinical Trial Registry (number: ChiCTR2300068970). The confidentiality and anonymity of participant data will be assured throughout the entire process, including during the implementation of the experimental protocol, as well as for any subsequent presentations or publications stemming from the study. Prior to the study, a participant information sheet will be provided to the participants, and their written informed consent will be sought to ensure compliance with ethical standards. Additionally, if any participant exhibits symptoms indicative of depression risk during our study, our trained research team will immediately conduct a thorough assessment. Based on this assessment, we will refer the participant to a qualified psychologist or another mental health specialist for further evaluation and, if necessary, professional intervention.

## 3 Discussion

College students are at a high risk of StD. Compared with young people without depressive symptoms, individuals with StD have an increased risk of developing MDD, and more adaptive dysfunction at home, community, and school (47). If successful, our program could provide an interesting and easy-to-use intervention for mental health issues that could benefit young people with StD. At the same time, exploring college students’ experiences of participating in this type of activities is helpful to further improve and promote exergame interventions.

Exercise therapy is characterized by low side effects, low cost and rapid effectiveness, and has been proven to be effective in alleviating depressive symptoms of individuals with StD (18, 48). As a form of exercise therapy, exergames make traditional exercise more interesting, interactive and enjoyable, improving the motivation and compliance of patients to participate in sports. A recent systematic review and meta-analysis showed that exergames help improving depressive symptoms in adults (19). Another study showed that exergames help improving depressive symptoms also in older adults (49). In the present study, our exergame intervention may lead to an improvement of depressive symptoms in college students, which may be the result of multiple factors. First, exergame interventions can provide participants with a variety of scenes, sounds, visual effects and other rich environmental stimulation through the construction of virtual environments. This creates an immersive experience, and increases the participants’ sense of enjoyment and engagement (50). This rich environmental stimulation can lead to changes in the endocrine system, such as promoting the secretion of adrenaline and cortisol, thereby reducing depressive symptoms and promoting emotional balance (51, 52). In addition, the multi-domain tasks set in the game can also bring more targeted stimulus information, so that participants get comprehensive sensory stimulation (53). This full range of sensory stimulation may help distract attention, shift emotions, and positively affect the brain’s cognitive and emotional processing (53). On the one hand, college students’ participation in exergame training continuously stimulates the excitability of the brain, and individual connections in the brain are constantly removed or recreated, causing neuroplasticity changes in the brain (54, 55), especially in the brain’s reward circuits and emotion regulation regions, helping to establish healthier neural network patterns (56). On the other hand, exercise training can promote the activity of the nervous system (51) and increase the release of neurotransmitters such as dopamine (57) and endorphins (58). These neurotransmitters are involved in feelings of pleasure, reward mechanisms, and mood regulation, and help improve depressive symptoms.

The timeframe of the exergame intervention is also a point of interest. A study conducted by Plotnikoff et al. found that interventions lasting a university semester or less (≤12 weeks) generally resulted in a greater number of significant outcomes (59), compared to interventions lasting more than a semester. In addition, a meta-analysis found that exergame interventions lasting between 6 and 12 weeks (SMD = −0.61, *p* = 0.042) had a higher effect on depression in adults than interventions lasting 6 weeks or less (SMD = −0.58, *p* < 0.001) (19). For this reason, it is necessary for researchers to find a balanced duration that maintains participant engagement while minimizing potential fatigue or boredom. Notably, some mental health exergame interventions lasting 8 weeks have been shown to be effective in improving depressive symptoms (60–62), which suggests this intervention duration may allow participants to fully engage in the program, experience changes, and demonstrate meaningful outcomes. For this reason, we chose an 8-week intervention duration. In addition, some studies have shown that training frequency has also an impact on the improvement of depressive symptoms (63). A recent meta-analysis showed that an increasing exercise frequency was associated with an increased improvement of depressive symptoms (64). The World Health Organization (WHO) (65) recommends engaging in muscle-strengthening activities at moderate or greater intensity on 2 or more days a week. Considering the above, in our study, intervention sessions took place 2–3 times per week.

However, this study will also face some challenges. First, the busy schedules and academic pressures of college students may affect their willingness to participate in the study in the first place, and for this reason, we will design attractive promotional materials that emphasize the importance and potential benefits of the study. To the best of our ability, we will provide appropriate incentives, such as awards or credits, to increase their willingness to participate. Secondly, the ability of participants to maintain long-term engagement and motivation in the game is a potential problem, which may affect the therapeutic effects of the intervention. For this purpose, the researchers will provide appropriate support and guidance, depending on the situation, to encourage participants to maintain long-term engagement and motivation in the game, such as setting goals or positive interactions. Thirdly, the consistency of the data may be compromised as participants may use the game in different environments. Therefore, clear guidelines for data collection will be provided to ensure that participants are held to the same standards each time they use the exergame. Finally, the scales used are self-reported measures, which can be subject to bias. Factors such as acute illness, changes in physical function, and stressful events can impact the accuracy of participants’ responses (66). Therefore, if conditions permit, objective indicators, such as physiological indicators, will be considered alongside self-reported measures, to reduce the bias of individual subjective feelings and improve the objectivity of the assessment.

## 4 Conclusion

Compared to the control group, college students with StD who receive the exergame intervention may show improvements in depressive and anxiety symptoms, subjective experience, sleep quality, and quality of life. If proven effective, exergame interventions will be easy to adhere to and implement. Additionally, the findings from the qualitative study will provide valuable insights to enhance the intervention’s effectiveness, adaptability, and overall satisfaction for individuals with StD.

## Ethics statement

The studies involving humans were approved by the institutional review board of each site (School of Nursing, Jilin University Clinical Research Ethics Committee) approved the protocol (number: 2022091401). The studies were conducted in accordance with the local legislation and institutional requirements. Written informed consent for participation in this study was provided by the participants’ legal guardians/next of kin.

## Author contributions

HKX: Conceptualization, Data curation, Formal analysis, Investigation, Methodology, Project administration, Resources, Software, Supervision, Validation, Visualization, Writing – original draft, Writing – review & editing. ML: Conceptualization, Data curation, Formal analysis, Investigation, Methodology, Project administration, Resources, Software, Supervision, Validation, Visualization, Writing – original draft, Writing – review & editing. YSM: Conceptualization, Data curation, Investigation, Methodology, Software, Writing – original draft, Writing – review & editing. JYL: Conceptualization, Formal analysis, Investigation, Methodology, Software, Writing – original draft. HRD: Conceptualization, Data curation, Formal analysis, Methodology, Validation, Writing – original draft. HBY: Conceptualization, Data curation, Formal analysis, Investigation, Methodology, Software, Writing – original draft, Writing – review & editing. PYH: Conceptualization, Formal analysis, Methodology, Software, Writing – original draft, Writing – review & editing. YJ: Funding acquisition, Supervision, Writing – original draft, Writing – review & editing. CL: Supervision, Writing – original draft, Writing – review & editing. SY: Conceptualization, Data curation, Investigation, Methodology, Software, Writing – original draft, Writing – review & editing.
