# Supplementary material for: The effectiveness of an exergame intervention for college students with subthreshold depression: protocol for a mixed methods study
Source: Front Public Health. 2024 Sep 2;12:1390806. doi: 10.3389/fpubh.2024.1390806 (PMC11402669; doi:10.3389/fpubh.2024.1390806)
Supplement: Supplementary file 1 [file Data_Sheet_1.docx]

**Supplementary Material**

**The Effectiveness of an Exergame Intervention for College Students with Subthreshold Depression: Protocol for a Mixed Methods Study**

Contents

Table S1. The Nintendo Switch-based exergame combination programme (Test version) 2

Table S2. The outline of the Favourite Exergame Questionnaire (FEQ) 3

Table S3. The Nintendo Switch-based exergame combination programme (First version) 4

Table S4. The Nintendo Switch-based exergame combination programme (Final version). 8

Table S5. Three stages of exergame intervention programme 11

Figure. S1. Details of Nintendo Switch-based exergame combination programme (Take the first week as an example) 12

Figure. S2. Flow chart of assessment 13

**Table S1.** The Nintendo Switch-based exergame combination programme (Test version).

|  | | **Intervention content** | | | **Duration** |
| --- | --- | --- | --- | --- | --- |
| Week 1 | Warm-up | |  | Easy Mode (optional) | 5.5 mins |
|  | Exergame | | Match1^a^ | Adventure Mode (optional) | 30-40 mins |
|  |  |  | Match2^b^ | Fun Games (optional) | 10 mins |
|  | Cool-down | |  | Stretching Exercises (optional) | 4.5 mins |
| Week 2 | Warm-up | |  | Dynamic stretch  Arm stretch  Abdominal stretch  Leg stretch  Yoga stretch | 5.5 mins |
|  | Exergame | | Match1^a^ | Adventure Mode | 30-40 mins |
|  |  |  | Match2^b^ | Chest training  Whack-a-mole  Parachute  Squat bounce  Land of Ichino | 10 mins |
|  | Cool-down | |  | Stretching exercises^c^ | 4.5 mins |

Note.

^a^ After each workout, keep a record, and finally accumulate and record the total score.

^b^ Choose 1-2 challenges provided for completion.

^c^ Stretching exercises focus on relaxing the abdominal, lumbar, and leg areas.

Abbr. mins, minutes.

**Table S2.** The outline of the Favourite Exergame Questionnaire (FEQ).

| **Name of the participant** | **Liking degree** | | | | |
| --- | --- | --- | --- | --- | --- |
|  | **1** | **2** | **3** | **4** | **5** |
| XXX |  |  |  |  |  |
| ……. |  |  |  |  |  |

**Table S3.** The Nintendo Switch-based exergame combination programme (First version).

| **Intervention time** | | **Intervention content** | | | **Duration** |
| --- | --- | --- | --- | --- | --- |
| Week 1 | Warm-up | |  | Dynamic stretch  Arm stretch  Abdominal stretch  Leg stretch  Yoga stretch | 5.5 mins |
|  | Exergame | | Match1^a^ | Adventure mode | 30-40 mins |
|  |  |  | Match2^b^ | Chest muscle training  Whack-a-mole  Parachuting  Squat jumps  Icino land | 10 mins |
|  | Cool-down | |  | Stretching exercises^c^ | 4.5 mins |
| Week 2 | Warm-up | |  | Dynamic stretch  Arm stretch  Abdominal stretch  Leg stretch  Yoga stretch | 5.5 mins |
|  | Exergame | | Match1^a^ | Adventure mode | 30-40 mins |
|  |  |  | Match2^b^ | Latissimus dorsi challenge  Squat jumps  Box breaking  Balance walking  Spada highway challenge | 10 mins |
|  | Cool-down | |  | Stretching exercises^c^ | 4.5 mins |
| Week 3 | Warm-up | |  | Dynamic stretch  Arm stretch  Abdominal stretch  Leg stretch  Yoga stretch | 5.5 mins |
|  | Exergame | | Match1^a^ | Adventure mode | 30-40 mins |
|  |  |  | Match2^b^ | Latissimus dorsi training  Quadriceps training  Whack-a-mole  Parachuting  Floating world hall | 10 mins |
|  | Cool-down | |  | Stretching exercises^c^ | 4.5 mins |
| Week 4 | Warm-up | |  | Dynamic stretch  Arm stretch  Abdominal stretch  Leg stretch  Yoga stretch | 5.5 mins |
|  | Exergame | | Match1^a^ | Adventure mode | 30-40 mins |
|  |  |  | Match2^b^ | Erector spinae training  Squat jumps  Inner thigh knight  High raise arms get  Little bridge trail | 10 mins |
|  | Cool-down | |  | Stretching exercises^c^ | 4.5 mins |
| Week 5 | Warm-up | |  | Dynamic stretch  Arm stretch  Abdominal stretch  Leg stretch  Yoga stretch | 5.5 mins |
|  | Exergame | | Match1^a^ | Adventure mode | 30-40 mins |
|  |  |  | Match2^b^ | Infinite challenge of deltoids  Arm pulling embryo  Inner thigh knight  Coin run  Running bridge | 10 mins |
|  | Cool-down | |  | Stretching exercises^c^ | 4.5 mins |
| Week 6 | Warm-up | |  | Dynamic stretch  Arm stretch  Abdominal stretch  Leg stretch  Yoga stretch | 5.5 mins |
|  | Exergame | | Match1^a^ | Adventure mode | 30-40 mins |
|  |  |  | Match2^b^ | Back latissimus endless challenge  Hand pulling embryo  Waist twisting strike  Coin running  Residence of the warcraft | 10 mins |
|  | Cool-down | |  | Stretching exercises^c^ | 4.5 mins |
| Week 7 | Warm-up | |  | Dynamic stretch  Arm stretch  Abdominal stretch  Leg stretch  Yoga stretch | 5.5 mins |
|  | Exergame | | Match1^a^ | Adventure mode | 30-40 mins |
|  |  |  | Match2^b^ | Back latissimus endless challenge  Hand pulling embryo  Waist twisting strike  Coin running  Residence of the warcraft | 10 mins |
|  | Cool-down | |  | Stretching exercises^c^ | 4.5 mins |
| Week 8 | Warm-up | |  | Dynamic stretch  Arm stretch  Abdominal stretch  Leg stretch  Yoga stretch | 5.5 mins |
|  | Exergame | | Match1^a^ | Adventure mode | 30-40 mins |
|  |  |  | Match2^b^ | Pectoralis maximus challenge  Parachute  Box break  Disc strike  Spoda highway | 10 mins |
|  | Cool-down | |  | Stretching exercises^c^ | 4.5 mins |

Note.

^a^ After each workout, keep a record, and finally accumulate and record the total score.

^b^ Choose 1-2 challenges provided for completion.

^c^ Stretching exercises focus on relaxing the abdominal, lumbar, and leg areas.

Abbr. mins, minutes.

**Table S4.** The Nintendo Switch-based exergame combination programme (Final version)

|  | | **Intervention content** | | | **Duration** |
| --- | --- | --- | --- | --- | --- |
| Week 2 | Warm-up | |  | Dynamic stretch  Arm stretch (Raise both arms forward)  Abdominal stretch (Swing arms, raise both arms sideways)  Leg stretch (Step, squat)  Yoga stretch (Twisting triangle pose) | 5.5 mins |
|  | Exergame | | Match1^a^ | Adventure mode | 30-40 mins |
|  |  |  | Match2^b^ | Latissimus dorsi challenge  Squat jumps  Box breaking  Balance walking  Spada highway challenge | 10 mins |
|  | Cool-down | |  | Stretching exercises^c^ | 4.5 mins |
| Week 3 | Warm-up | |  | Dynamic stretch  Arm stretch (Twist arms)  Abdominal stretch (Triceps, Raise arms and side bend)  Leg stretch (Continuous leg lifts, Butterfly pose)  Yoga stretch (Raise arms morning pose) | 5.5 mins |
|  | Exergame | | Match1^a^ | Adventure mode | 30-40 mins |
|  |  |  | Match2^b^ | Latissimus dorsi training  Quadriceps training  Whack-a-mole  Parachuting  Floating world hall | 10 mins |
|  | Cool-down | |  | Stretching exercises^c^ | 4.5 mins |
| Week 4 | Warm-up | |  | Dynamic stretch  Arm stretch (Twisting arms)  Abdominal stretch (Triceps, Side bend with raised arms)  Leg stretch (Continuous Leg Lifts, Plie Pose)  Yoga stretch (Raised arms morning pose) | 5.5 mins |
|  | Exergame | | Match1^a^ | Adventure mode | 30-40 mins |
|  |  |  | Match2^b^ | Erector spinae training  Squat jumps  Inner thigh knight  High raise arms get  Little bridge trail | 10 mins |
|  | Cool-down | |  | Stretching exercises^c^ | 4.5 mins |
| Week 5 | Warm-up | |  | Dynamic stretch  Arm stretching (Twisting arms, Hero 2 pose)  Abdominal stretching (Bent-over rowing)  Leg stretching (Stepping, lifting thighs)  Yoga stretching (Morning pose with raised arms) | 5.5 mins |
|  | Exergame | | Match1^a^ | Adventure mode | 30-40 mins |
|  |  |  | Match2^b^ | Infinite challenge of deltoids  Arm pulling embryo  Inner thigh knight  Coin run  Running bridge | 10 mins |
|  | Cool-down | |  | Stretching exercises^c^ | 4.5 mins |
| Week 6 | Warm-up | |  | Dynamic stretch  Arm stretching (Twist arms, Hero 2 pose)  Abdominal stretching (Bent-over rowing)  Leg stretching (Step-up, Lift thighs)  Yoga stretching (Raised arms morning pose | 5.5 mins |
|  | Exergame | | Match1^a^ | Adventure mode | 30-40 mins |
|  |  |  | Match2^b^ | Back latissimus endless challenge  Hand pulling embryo  Waist twisting strike  Coin running  Residence of the warcraft | 10 mins |
|  | Cool-down | |  | Stretching exercises^c^ | 4.5 mins |
| Week 7 | Warm-up | |  | Dynamic stretch  Arm stretch (shoulder push, arms twisting)  Belly stretch  Leg stretch (wide squat, chair position)  Yoga stretch (Mountaineering) | 5.5 mins |
|  | Exergame | | Match1^a^ | Adventure mode | 30-40 mins |
|  |  |  | Match2^b^ | Quadriceps challenge  Climb  Disc strike  Box break  Land of ichino | 10 mins |
|  | Cool-down | |  | Stretching exercises^c^ | 4.5 mins |
| Week 8 | Warm-up | |  | Dynamic stretch  Arm stretch (shoulder push, arms twisting)  Belly stretch  Leg stretch (wide squat, chair position)  Yoga stretch (Mountaineering) | 5.5 mins |
|  | Exergame | | Match1^a^ | Adventure mode | 30-40 mins |
|  |  |  | Match2^b^ | Pectoralis maximus challenge  Parachute  Box break  Disc strike  Spoda highway | 10 mins |
|  | Cool-down | |  | Stretching exercises^c^ | 4.5 mins |

Note.

^a^ After each workout, keep a record, and finally accumulate and record the total score.

^b^ Choose 1-2 challenges provided for completion.

^c^ Stretching exercises focus on relaxing the abdominal, lumbar, and leg areas.

Abbr. mins, minutes.

**Table S5.** The three stages of the exergame intervention programme.

| **EI** | **Aim** | **Duration** |
| --- | --- | --- |
| Warm-up | Muscle relaxation | 5.5min |
| Exergame | Match1 and Match2 | 40-50 min |
| Cool-down | Stretching exercise | 4.5min |

Notes. The “Warm-up” will consist of five parts: “dynamic stretch”, “arm stretch”, “abdominal stretch”, “leg stretch”, and “yoga stretch” (custom mode), and the whole process will last approximately 5.5 minutes. The “Exergame” will include two parts: “Match1” and “Match2”. The “Match1” will correspond the “adventure mode”, in which players will be required to defeat the enemies using a combination of different physical activities, which will last 30-40 minutes. The “Match2” will be a single challenge, and lasts 10 minutes (choose one or two games). The “Cool-down” section lasts about 4.5 minutes. More details are provided in Figure S1.

**Figure. S1**. Details of the Nintendo Switch-based exergame combination programme (Week 1 example).


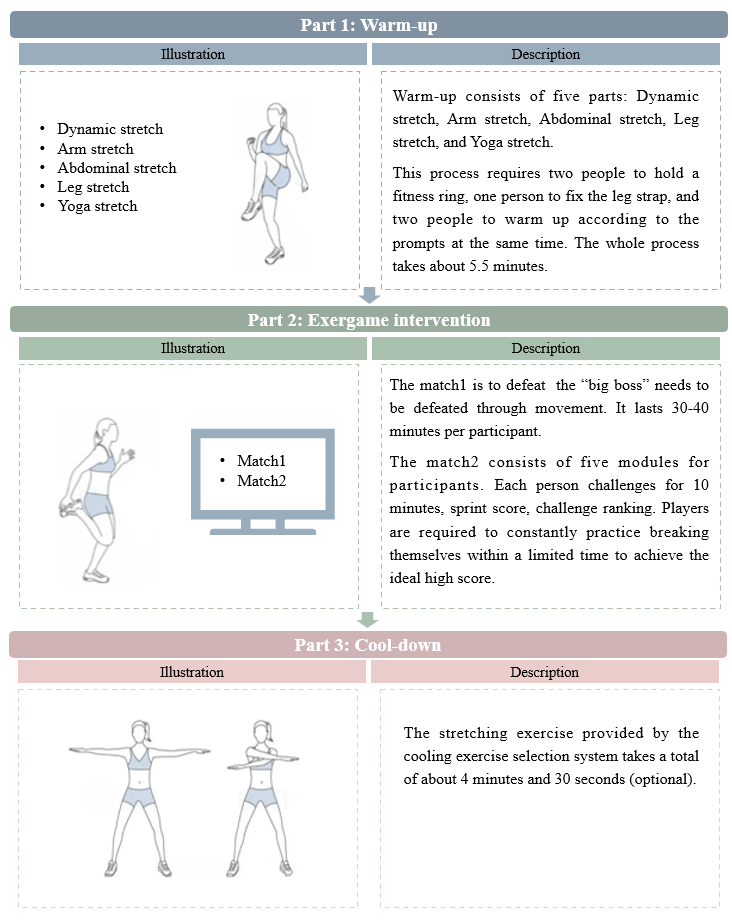


**Figure. S1**. Details of the EI training programme (Week 1 example).

**Figure. S2**. Flow chart of the assessment.


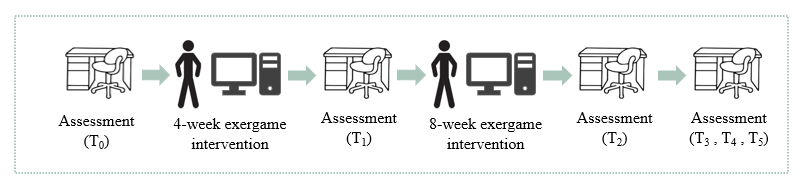


**Figure. S2.** Flow chart of the assessment.
